# Medication reconciliation on discharge in a tertiary care Riyadh Hospital: An observational study

**DOI:** 10.1371/journal.pone.0265042

**Published:** 2022-03-15

**Authors:** Ahmed S. Alanazi, Sameh Awwad, Tahir M. Khan, Syed Mohammed Basheeruddin Asdaq, Yahya Mohzari, Foz Alanazi, Ahmed Alrashed, Abdulhakeem S. Alamri, Walaa F. Alsanie, Majid Alhomrani, Mohammed AlMotairi

**Affiliations:** 1 Pharmaceutical Service Department, Main Hospital, King Fahad Medical City, Riyadh, Saudi Arabia; 2 The Institute of Pharmaceutical Science (IPS) of University of Veterinary and Animal Sciences, Lahore, Pakistan; 3 Department of Pharmacy Practice, College of Pharmacy, AlMaarefa University, Riyadh, Saudi Arabia; 4 Pharmacy Department, Clinical Pharmacy Section, King Saudi Medical City, Riyadh, Saudi Arabia; 5 Cardiac Center, Ministry of National Guard Health Affairs, Riyadh, Saudi Arabia; 6 Department of Clinical Laboratory Sciences, The Faculty of Applied Medical Sciences, Taif University, Taif, Saudi Arabia; 7 Centre of Biomedical Sciences Research (CBSR), Deanship of Scientific Research, Taif University, Taif, Saudi Arabia; 8 Department of Clinical Pharmacy, King Fahad Medical City, Riyadh, Saudi Arabia; Nobel College Pvt Ltd, NEPAL

## Abstract

The purpose of this study was to assess the frequency and characteristics of discharge medication discrepancies as identified by pharmacists during discharge medication reconciliation. We also attempted to identify the factors that influence the occurrence of drug discrepancies during medication reconciliation. From June to December 2019, a prospective study was performed at the cardiac center of King Fahad Medical City (KFMC), a tertiary care hospital in Riyadh. The information from discharge prescriptions as compared to the medication administration record (MAR), medication history in the cortex system, and the patient home medication list collected from the medication reconciliation form on admission. The study included all adult patients discharged from KFMC’s cardiac center. These participants comprised 776 patients, 64.6 percent of whom were men and 35.4 percent of whom were women. Medication discrepancies were encountered in 180 patients (23.2%) out of 776 patients. In regards to the number of discharged medications, 651(83.9%) patients had ≥ 5 medications. Around, 174 (73.4%) discrepancies were intentional, and 63 (26.6%) were unintentional discrepancies. The risk of unintentional medication discrepancy was increased with an increasing number of medications (P-value = 0.008). One out of every four cardiac patients discharged from our hospital had at least one medication discrepancy. The number of drugs taken and the number of discrepancies was found to be related. Necessary steps should be taken to reduce these discrepancies and improve the standard of care.

## Introduction

A process or procedure used by health care providers to obtain a full and correct list of a patient’s prescription and non-prescribed medications is known as Medication reconciliation. It is described by the Agency for Healthcare Research and Quality (AHRQ) as the method or technique used by health care providers to compile a full and accurate medication list for a patient’s prescription and home medications [1]. Medication reconciliation in clinical practice is considered an essential step to ensure patient safety and to avoid discrepancies or inconsistencies in patient’s medications at the time of admission [2] or transition from one unit to another or at the time of discharge [3]. Elderly patients with a severe condition, are at high risk for medication discrepancies due to the multiple re-admission, and change in drug generics [4]. Also, the complexity of the regimen has been associated with an increase in medication discrepancies [5, 6]. At the time of discharge, the most common types of discrepancies were incomplete, inaccurate, illegible discharge instructions, and drug omission [7, 8]. The Joint Commission [9] has designated medication reconciliation as a top priority for national patient safety targets. Medication reconciliation has also been recommended by the Institute of Healthcare Improvement as a validated method to reduce adverse drug effects due to medication discrepancies [10].

Medication reconciliation at King Fahad Medical City (KFMC) cardiac unit is a manual and paper-based process, which adds to the increased risk of medication errors. In Saudi Arabia, it is observed that 70% of medical ward patients were found to be affected by these medication discrepancies and about 18% of them had at least one unintentional discrepancy [11]. Another study showed that medication discrepancies at admission were estimated to be around 37% [12]. However, a prospective, controlled study showed that clinical implementation of medication reconciliation doesn’t affect re-hospitalization or emergency re-admissions and mortality over 6 months follow up [13, 14]. Additionally, implementation of medication reconciliation service at admission, a transition of care and discharge has reduced medication discrepancies in adult and pediatric cardiac surgery patients [6].

In an established tertiary care setting, the pharmacist plays a vital role to minimize the chances of discrepancies at admission, transfer out and discharge [6]. The role of pharmacist in regulating the flow of medications and restriction of medication errors is well documented [15]. Therefore, this study was conducted at the cardiac center of KFMC in Saudi Arabia to determine the frequency and characteristics of discharge medication discrepancies discovered by pharmacists during discharge medication reconciliation.

## Materials and methods

### Data collection

This prospective observational study was conducted in tertiary specialty referral hospitals known as KFMC in Riyadh, Saudi Arabia. Over 6 months’ period between 15/6/2019 to 15/12/2019, all patients discharged from king Salman’s cardiac care center of the hospital were included in the analysis. All prescriptions submitted electronically to the discharge pharmacy for patients who were expected to be discharged were analyzed. The medications were then cross-referenced with the patient’s home medication list obtained from the emergency room’s medication reconciliation form during admission, as well as the medication administration record (MAR) and medication history in the Cortex system (clinical management software).

### Data screening

A team of qualified pharmacists who were trained in medication reconciliation manually extracted the data of each patient at the time of discharge. The information obtained was age, gender, number of medications, and class of the medications. Further, they determined discrepancies status to know whether there exists a discrepancy. A discharge medication discrepancy was defined as any difference found between the medications listed on the discharge prescription and the medications listed in the patient’s history, Medication Administration Record (MAR), or home medications list [12]. Discrepancies were classified as either intended or unintended, and then further classified as omission, commission, changed dose, frequency, or route, incorrect duration or quantity, and therapeutic duplication. The severity of discrepancies, category of discrepancies, type of discrepancies, and number of discrepancies per patient were also explored. In the event of doubt, the prescriber was consulted. Additionally, the severity of discrepancies, category of discrepancies, type of discrepancies, and number of discrepancies per patient were analyzed.

The severity of medication discrepancies was classified into 3 categories [16]; Minor: in which omitted medication would not affect the patient’s outcome. Moderate: if this discrepancy continued or was received by the patient, it would have an undesirable effect on the patient’s outcome. Major: if this discrepancy continued or received by the patient would result in injury to the patient’s outcome. Finally, clinical records of patients were reviewed to determine whether the discrepancies found were intentional or unintentional. An intentional discrepancy is one that the prescriber made intentionally to meet the patient’s need, with documentation of the intended change in the clinical record, whereas an unintentional discrepancy is one that the physician or pharmacist made accidentally and/or did not document in the clinical records.

### Ethical consideration

Research center at King Fahad medical City and AlMaarefa University granted ethical approval vide reference number 18–454 and MCST (AU)-COP 1932/RC, respectively, to do this research. The data was obtained in an entirely anonymous manner. Informed consent was waived by the ethical committee as the study did not pose any risk to the participants.

### Statistical analysis

The average number of discrepancies per patient as well as the percentage of patients with at least one unintentional discrepancy were recorded. Besides that, the characteristics of identified medication discrepancies were examined, including the severity, frequency, and percentage of each discrepancy category. All categorical variables are described using frequency and percentage. To investigate the relationship between the categorical variables, the Chi-square test was used. A P-value less than 0.05 is regarded as statistically significant. The statistical package SPSS 22 was used to enter and analyze all of the data (SPSS Inc., Chicago, IL, USA).

## Results

A total of 776 patients were included in this study, the mean age of the patients was 58.34 ± SD13.71. Baseline characteristics of patients were described in Table 1. Medication discrepancies were found in 180 patients (23.2%) out of 776 patients. Of which 237 medication discrepancies were caught. The frequency of medication discrepancies is inconsistent with each prescription in which 42 (23.3%) patients were found to have more than 1 medication discrepancies. After medication review, 63 (26.6%) of medication discrepancies were categorized as unintended medication discrepancies where 174 (73.4%) were intentional and appropriately discharged on different medications/doses/frequencies as part of the new discharge plan. As for the number of medications, 651 (83.9%) of prescriptions had ≥5 discharge medications labeled with medication discrepancy following implementing medication reconciliation. The most common class of medications involved in discrepancies were Insulin, antiplatelet (Aspirin and Clopidogrel), beta-blocker, and statin and diuretics, accounting for 14.3%, 12.7%, 9.5%, and 7.9%, respectively.

**Table 1:** Demographic and clinical characteristics of patients (n = 776).

Characteristics	Descriptions	N (%)
**Age**	Mean ± SD	58.34 ± 13.71
**Gender**	Male	502 (64.7%)
	Female	274 (35.3%)
**Number of medications**	Less than 5	125 (16.1%)
	5 or more	651 (83.9%)
**Discrepancy status**	Yes	180 (23.2%)
	No	596 (76.8%)

Data given as mean±SD.

Table 2 revealed the discrepancies of the other classes of medications found in this study. Of the 63 unintentional medication discrepancies identified during patient discharge, the most type of medication discrepancy was an omission of medication by 21 (33.3%), followed by dose changed by 18 (28.6%), (Table 2). The severity of discrepancies encountered was primarily classified as major and moderate accounting for over 62 (98%) of unintended discrepancies (Table 2).

**Table 2:** Clinical characteristics of patients.

**Number of discrepancies per patient**	1 Discrepancy per patient	138 (58.2%)
	2 Discrepancies per patient	58 (24.5%)
	≥3 Discrepancies per patient	41 (17.3%)
**Category of discrepancy**	Intended	174 (73.4%)
	Un-intended	63 (26.6%)
**Type of un-intentional discrepancy**	Omission	21 (33.3%)
	Changed Dose	18 (28.6%)
	Changed Frequency	13 (20.6%)
	Commission	5 (7.9%)
	Wrong Duration	4 (6.3%)
	Changed Route	2 (3.2%)
**Severity of un-intentional discrepancy**	Major	24 (38.1%)
	Minor	1 (1.6%)
	Moderate	38 (60.3%)
**Class of Medication found with un-intentional discrepancy**	Insulins	9 (14.3%)
	Anti-Platelet	8 (12.7%)
	Beta-Blocker	6 (9.5%)
	Antimicrobial Medications	5 (7.9%)
	Diuretics	5 (7.9%)
	Statins	5 (7.9%)
	Anti-Coagulation	4 (6.4%)
	Oral Hypoglycemic Medications	4 (6.3%)
	Calcium Chanel Blocker’s	3 (4.8%)
	Others	14 (22.3%)

Data given as mean±SD.

Unintentional drug discrepancies were linked to an increased number of drugs (P-value = 0.008). (Table 3). On the other hand, older age did not affect drug discrepancy (P-value = 0.259).

**Table 3:** Association between the unintentional discrepancy of the medication and study characteristics.

Characteristics	Descriptions	Discrepancy		P—value
		Yes	No	
**Age Group**	<=30	3 (4.8%)	12 (2.0%)	0.259
	31–50	10 (15.9%)	126 (21.1%)	
	> 50	50 (79.4%)	458 (76.8%)	
**Medications**	< 5 Medications	0 (0.0%)	60 (10.1%)	*0.008
	>= 5 Medications	63 (100.0%)	536 (89.9%)	

Note: A P-value less than 0.05 is considered statistically significant.

## Discussion

In hospitalized patients, the drug reconciliation procedure is an effective way to minimize unintended medication discrepancies and medication errors [17]. At our hospital medication reconciliation process was implemented in the emergency room (ER) during admission that may lead to a decrease in the incidence of medication discrepancy than hospitals that didn’t have medication reconciliation at admission or during the transition of care and discharge.

Our results are in line with the previous study, which found a 30% incidence of medication discrepancy [12, 18]. Mekonnen AB et al. [17] and Mazhar et al. [18] found that omission was the most common form of medication discrepancy, which is consistent with our findings. Our findings were in line with those of Mazhar et al. [18] who found that polypharmacy was linked to more drug discrepancies. Other reports, on the other hand, found the reverse in the case of polypharmacy [16, 17].

There was no significant association between increased patient age and medication discrepancies. Since the majority of the patients in this study were over the age of 50, an equally large number of older patients were found in both with and without discrepancy groups (Table 3). Although we included all discharge patients during the study period, it appears that most of our admitted patients in the cardiology center were older than 50 years age. Overall, the findings of this study are consistent with earlier reports in which patients over the age of 65 are documented to have improved compliance with antihypertensive therapy and congestive heart failure care [19].

Our results were consistent with Grimes, T.C. et al. [20] which found that most of the medication discrepancy encountered were moderate- minor in severity. The strength of our study was that we included all patients admitted to the cardiology ward for 6 months, which amounted to approximately 800 patients. We have conducted a prospective observational study rather than a retrospective. The current literature on drug usage and abuse has concentrated mainly on one type of discrepancy, namely patient compliance, which tests patients’ inability to take their drugs as prescribed. Our research emphasizes the broader picture of discrepancy and builds on Wagner and Hogan’s previous work [21], showing that a patient’s drug choice is not purely dependent on their willingness. Other studies indicate that other factors, such as miscommunication among physicians or between physicians and patients, may play a significant role [22, 23]. When our patients refused to take prescribed medications or took additional non-recorded drugs, they were often following another physician’s instructions, which happened outside of our clinical practice.

This observational research has some limitations. First, although the study sample was representative of KFMC’s cardiac center as a whole, our findings may not be relevant to other clinical settings or patients participating in other hospitals in the area because many do not adhere to the drug reconciliation process. Nonetheless, it is vital to note that health facilities across the country with similar medication reconciliation processes may have a nearly identical amount of discrepancies due to the same medical practice and patients’ attitudes toward prescription storage at home. Second, the effect of medication discrepancy on patient outcomes was not examined in this study. Third, we haven’t measured any cost-effectiveness analysis which would have to strengthen the outcome evaluation. Finally, our study was an observational study rather than an interventional randomized study which increases the risk of confounders, and bias that can’t be controlled.

## Conclusions

It is important to focus on reducing drug discrepancies. The use of a standardized drug questionnaire and the establishment of a telecommunication system for monitoring drugs with the help of a pharmacist can aid in the monitoring of medication use. We hope to start a program of medication reconciliation and monitoring, particularly for patients who are chronically ill and have multiple morbidities.

One out of every four cardiac patients discharged from our hospital had at least one drug discrepancy. An increased number of prescriptions was linked to the incidence of prescription discrepancies at discharge. Major severity of medication discrepancy found in one-third of our patient who experiences an unintended medication discrepancy which mandates the need for medication reconciliation process to avoid such medication error. A study with a larger sample size will be more reliable and validate these findings. Nevertheless, necessary steps should be taken to reduce these discrepancies and improve the standard of care.
