# Correction: Medication reconciliation on discharge in a tertiary care Riyadh Hospital: An observational study

**DOI:** 10.1371/journal.pone.0286177

**Published:** 2023-05-18

**Authors:** Ahmed S. Alanazi, Sameh Awwad, Tahir M. Khan, Syed Mohammed Basheeruddin Asdaq, Yahya Mohzari, Foz Alanazi, Ahmed Alrashed, Abdulhakeem S. Alamri, Walaa F. Alsanie, Majid Alhomrani, Mohammed AlMotairi

The affiliations for the second and eleventh authors are incorrect. Sameh Awwad is not affiliated with #2 and Mohammed AlMotairi not affiliated with #8. Both authors are affiliated with #1: Pharmaceutical Service Department, Main Hospital, King Fahad Medical City, Riyadh, Saudi Arabia.
